# Prevalence and Prognosis of Lynch Syndrome and Sporadic Mismatch Repair Deficiency in Endometrial Cancer

**DOI:** 10.1093/jnci/djab029

**Published:** 2021-03-08

**Authors:** Cathalijne C B Post, Ellen Stelloo, Vincent T H B M Smit, Dina Ruano, Carli M Tops, Lisa Vermij, Tessa A Rutten, Ina M Jürgenliemk-Schulz, Ludy C H W Lutgens, Jan J Jobsen, Remi A Nout, Emma J Crosbie, Melanie E Powell, Linda Mileshkin, Alexandra Leary, Paul Bessette, Hein Putter, Stephanie M de Boer, Nanda Horeweg, Maartje Nielsen, Tom van Wezel, Tjalling Bosse, Carien L Creutzberg

**Affiliations:** 1 Department of Radiation Oncology, Leiden University Medical Center, Leiden, the Netherlands; 2 Department of Pathology, Leiden University Medical Center, Leiden, the Netherlands; 3 Department of Clinical Genetics, Leiden University Medical Center, Leiden, the Netherlands; 4 Department of Radiation Oncology, University Medical Center Utrecht, Utrecht, the Netherlands; 5 Department of Radiation Oncology, MAASTRO Clinic, Maastricht, the Netherlands; 6 Department of Radiation Oncology, Medical Spectrum Twente, Enschede, the Netherlands; 7 Division of Cancer Sciences, University of Manchester, St Mary’s Hospital, Manchester, UK; 8 Department of Obstetrics and Gynaecology, Manchester University NHS Foundation Trust, Manchester, UK; 9 Department of Clinical Oncology, Barts Health NHS Trust, London, UK; 10 Department of Medical Oncology, Peter MacCallum Cancer Centre, Melbourne, Australia; 11 Department of Medical Oncology, Gustave Roussy Cancer Center—INSERM U981, Université Paris Saclay, Villejuif, France; 12 Department of Obstetrics and Gynecology, University of Sherbrooke, Sherbrooke, Quebec, Canada; 13 Department of Biostatistics, Leiden University Medical Center, Leiden, the Netherlands

## Abstract

**Background:**

Standard screening of endometrial cancer (EC) for Lynch syndrome (LS) is gaining traction; however, the prognostic impact of an underlying hereditary etiology is unknown. We established the prevalence, prognosis, and subsequent primary cancer incidence of patients with LS-associated EC in relation to sporadic mismatch repair deficient (MMRd)-EC in the large combined Post Operative Radiation Therapy in Endometrial Carcinoma-1, -2, and -3 trial cohort.

**Methods:**

After MMR-immunohistochemistry, *MLH1*-promoter methylation testing, and next-generation sequencing, tumors were classified into 3 groups according to the molecular cause of their MMRd-EC. Kaplan-Meier method, log-rank test, and Cox model were used for survival analysis. Competing risk analysis was used to estimate the subsequent cancer probability. All statistical tests were 2-sided.

**Results:**

Among the 1336 ECs, 410 (30.7%) were MMRd. A total of 380 (92.7%) were fully triaged: 275 (72.4%) were *MLH1*-hypermethylated MMRd-ECs; 36 (9.5%) LS MMRd-ECs, and 69 (18.2%) MMRd-ECs due to other causes. Limiting screening of EC patients to 60 years or younger or to 70 years or younger would have resulted in missing 18 (50.0%) and 6 (16.7%) LS diagnoses, respectively. Five-year recurrence-free survival was 91.7% (95% confidence interval [CI] = 83.1% to 100%; hazard ratio = 0.45, 95% CI = 0.16 to 1.24, *P *=* *.12) for LS, 95.5% (95% CI = 90.7% to 100%; hazard ratio = 0.17, 95% CI = 0.05 to 0.55, *P *=* *.003) for “other” vs 78.6% (95% CI = 73.8% to 83.7%) for *MLH1*-hypermethylated MMRd-EC. The probability of subsequent LS-associated cancer at 10 years was 11.6% (95% CI = 0.0% to 24.7%), 1.5% (95% CI = 0.0% to 4.3%), and 7.0% (95% CI = 3.0% to 10.9%) within the LS, “other,” and *MLH1*-hypermethylated MMRd-EC groups, respectively.

**Conclusions:**

The LS prevalence in the Post Operative Radiation Therapy in Endometrial Carcinoma trial population was 2.8% and among MMRd-ECs was 9.5%. Patients with LS-associated ECs showed a trend towards better recurrence-free survival and higher risk for second cancers compared with patients with *MLH1*-hypermethylated MMRd-EC.

The diagnosis of Lynch syndrome (LS) in endometrial cancer (EC) is crucial for counseling and cancer surveillance of patients and their relatives. LS is a highly penetrant, hereditary, cancer-prone syndrome caused by germline variants in the DNA mismatch repair (MMR) genes: mutL homologue 1 (*MLH1*), mutS homologue 2 (*MSH2*), mutS homologue 6 (*MSH6*), or postmeiotic segregation increased 2 (*PMS2*). The cancer risk varies per gene and is substantially lower for *PMS2* (1,2). EC is often the first malignancy affecting women with LS (3), and their risk of metachronous cancer is approximately 24% at 10 years (4).

LS-associated cancers arise following MMR deficiency (MMRd) due to the somatic inactivation of the remaining wild-type *MMR* allele. MMRd leads to the accumulation of mismatches, insertions, and deletions in repeated sequences also known as microsatellite instability (MSI). MMRd is not an exclusive feature of LS; the vast majority (about 70%) of MMRd-ECs present with somatic inactivation of the *MLH1* gene via hypermethylation of the promoter region (5,6). Most of the cases that are neither *MLH1* hypermethylated nor harbor a MMR germline variant are considered sporadic due to biallelic somatic *MMR* gene inactivation; few are caused by an undetectable hereditary syndrome (frequently referred to as Lynch-like syndrome) (7-9). MMRd-ECs are known to have an intermediate prognosis within the molecular classification with a good response to immunotherapy (10‐13). The diagnosis of LS may allow clinicians to tailor treatment and patient information; LS-associated tumors may have a more favorable outcome (14), although there are no previous studies available on the prognostic impact of LS among MMRd-ECs.

Tumor triage by MMR-immunohistochemistry (IHC) and/or MSI analysis in combination with targeted *MLH1*-methylation testing can identify patients with LS. The Proportion of Endometrial Tumours Associated Lynch Syndrome study showed that IHC-based triage is most accurate, whereas clinical selection based on age and family history were imprecise predictors (15). Overall, an estimated 3% of EC cases are associated with LS (15‐17), which is similar in colorectal cancer (CRC) (18). However, these estimations were mostly based on small trials with methodological heterogeneity, often selecting their test population by age and/or family history, and incomplete testing (16).

Given its relative rarity, the prevalence and prognosis of LS should be investigated in a large population, such as the well-documented combined cohort of the Post Operative Radiation Therapy in Endometrial Carcinoma (PORTEC)-1, -2, and -3 trials. These randomized controlled trials have had a major impact on guidelines for treatment in ECs (19‐21). Together they included 1336 evaluable patients comprising all risk groups with long and complete follow-up information and collected tumor blocks. The aim of our study was to investigate the prevalence and prognosis of LS-associated EC in relation to *MLH1* hypermethylated MMRd-EC. Secondary objectives were to evaluate currently used age criteria for IHC-based tumor triage and the probability of developing a subsequent primary LS-associated cancer.

## Methods

### Study Population

In total, 1336 of 1801 ECs from the PORTEC-1, -2, and -3 clinical trials were eligible for analysis based on availability of formalin-fixed paraffin-embedded (FFPE) slides. In the PORTEC-1 trial (1990-1997), 714 patients with stage I low-intermediate and high-intermediate risk endometrioid EC were randomly assigned to receive pelvic radiotherapy or no additional treatment (19). In the PORTEC-2 trial (2002-2006), 427 endometrioid EC patients with high-intermediate risk features were randomly assigned to receive pelvic radiotherapy or vaginal brachytherapy (if stage I: ≥60 years) (20). In the international PORTEC-3 trial (2006-2013), 660 EC patients with high-risk features were randomly assigned to receive pelvic radiotherapy or chemoradiotherapy (21). In all trials, patients with a history of invasive cancer (for PORTEC-3 within the last 10 years), except for nonmelanoma skin cancer, were excluded. Full details and results of these trials have been published previously (19‐21). The study protocols were approved by the Dutch Cancer Society and the medical ethics committees at participating centers. All patients provided informed consent for participation in the trial, and for use of their tumor block for subsequent translational research. Clinicopathological data including p53-IHC and *POLE*-mutation status were obtained from the trial databases. Specific ethics approval was obtained for variant analysis on normal tissue among those suspected of LS. Cases from PORTEC-1 and -2 were analyzed anonymized in view of the long interval since recruitment. Cases from PORTEC-3 who were found to have LS were informed by their own physicians if LS had not been already diagnosed clinically. PORTEC-1 was conducted before time of trial registries. PORTEC-2 is registered with ISRCTN number ISRCTN16228756, and ClinicalTrials.gov number NCT00376844. PORTEC-3 is registered with ISRCTN number ISRCTN14387080, and ClinicalTrials.gov number NCT00411138.

### IHC, MSI, Methylation Analysis, and Next-Generation Sequencing (NGS)

Patients were included in the current analysis if they showed loss of expression of at least 1 of the 4 MMR proteins with positive internal control (including subclonal loss defined as abrupt and complete regional loss with intervening stromal positivity) or MSI-high status when MMR-IHC failed. Details on MMR-IHC and MSI testing and scoring were described previously (5,11,12,22). Cases with MMRd phenotype are referred to as MMRd-EC in this study irrespective of *POLE* mutation status.

*MLH1* methylation testing was performed on MLH1-deficient and/or MSI-high tumors as described previously (23). All cases with loss of MLH1 or MSI-high status without *MLH1* hypermethylation; loss of MSH2 and/or MSH6; or isolated loss of PMS2 were triaged as potential LS-associated MMRd-EC. DNA isolated from matched normal/tumor FFPE tissues of these cases was amplified using long-range polymerase chain reaction followed by targeted NGS for variants in the exonic regions of *MLH1*, *MSH2*, *MSH6*, *PMS2*, *POLE*, and *POLD1* using the Ion Proton System or Ion S5 System (Thermo Fisher Scientific) (24,25). Variants were annotated according to the following GenBank reference sequences: NM_000249.3 (*MLH1*), NM_000251.2 (*MSH2*), NM_000179.2 (*MSH6*), NM_000535.5 (*PMS2*), NM_006231.2 (*POLE*), and NM_001256849.1 (*POLD1*). All patients with germline variants (likely) affecting function (*path_MMR*) were verified by a clinical laboratory geneticist (C.M.T.) and considered to have LS.

### Statistical Analysis

Following complete triage, cases were classified into 3 groups according to the molecular cause of their MMRd-EC: LS, methylated (including cases with *MLH1* hypermethylation and subclonal MLH1 loss), and other causes (a mixed group having alternative causes of MMRd; see the Supplementary Methods and Supplementary Figure 1 for full definitions, available online). χ^2^ Statistics or Fisher’s exact test for categorical variables and 1-way analysis of variance or Kruskal-Wallis test for continuous variables were used to compare characteristics.

The sample size ensured sufficient power to detect an LS prevalence of 3.0% with a precision of 0.009 (95% confidence interval [CI] = 2.1% to 3.9%) within the whole population and a prevalence of 12.0% with a precision of 0.03 (95% CI = 9.0% to 15.0%) within the MMRd group (26).

Recurrence-free survival (RFS) was defined as time from random assignment to date of first relapse or death of any cause, whichever occurred first. Overall survival (OS) was defined as time from random assignment to date of death of any cause. Patients without an RFS or OS event were censored at the date of last contact. Five-year survival rates were estimated using the Kaplan-Meier method and compared with log-rank test. Cox proportional hazard models were used to estimate hazard ratios (HRs) over time; for adjusted analysis, age was included as covariate. The proportional hazard assumption was verified using Schoenfeld residuals. A competing-risk model with death as a competing event was used to estimate the cumulative incidence of developing a LS-associated second primary cancer (ie, colorectal, gallbladder, kidney, pancreas, small intestine, stomach, urinary bladder, and ureter cancer) in the different groups. A cause-specific Cox proportional hazard model was used to assess the statistical difference between the estimated probabilities. Time at risk started at random assignment and ended at date of occurrence of the first second cancer, death, or last date of study follow-up. *P* values less than .05 (2-tailed) were considered statistically significant. Statistical analyses were performed using R version 3.6.1.

## Results

### Study Population

Among the 1336 evaluable ECs, 410 (30.7%) were MMRd and eligible for further analysis. Median age of MMRd-EC patients was 65 years (interquartile range = 59-73 years). Most MMRd-ECs were early-stage tumors (74.2%) of low-grade endometrioid subtype (66.8%) and were treated with pelvic radiotherapy (51.7%). All characteristics of MMRd-ECs differed between the 3 PORTEC trials, in line with the inclusion criteria (Table 1).

**Table 1.** Patient, tumor, and treatment characteristics

Characteristic	All MMRd-EC	PORTEC-1	PORTEC-2	PORTEC-3	*P* ^a^
Total, No. (%)	410 (100.0)	145 (35.6)	114 (27.8)	151 (36.8)	
Age at random assignment					<.001
Median (IQR), y	65 (59-73)	67 (61-73)	70 (65-77)	60 (56-66)	
FIGO 2009 stage, No. (%)					<.001
IA	104 (25.4)	62 (42.8)	25 (21.9)	17 (11.3)	
IB	200 (48.8)	83 (57.2)	87 (76.3)	30 (19.9)	
II	36 (8.8)	0 (0.0)	1 (0.9)	35 (23.2)	
III	70 (17.1)	0 (0.0)	1 (0.9)	69 (45.7)	
Histological grade and type, No. (%)					<.001
EEC grade 1 or 2	274 (66.8)	122 (84.1)	91 (79.8)	61 (40.4)	
EEC grade 3	99 (24.1)	22 (15.2)	21 (18.4)	56 (37.1)	
Serous	11 (2.7)	1 (0.7)	2 (1.8)	8 (5.3)	
Clear cell	12 (2.9)	0 (0.0)	0 (0.0)	12 (7.9)	
Other	14 (3.4)	0 (0.0)	0 (0.0)	14 (9.3)	
Myometrial invasion, No. (%)					.001
≥50%	274 (66.8)	83 (57.2)	90 (78.9)	101 (66.9)	
Lymphovascular space invasion, No. (%)					<.001
Present	131 (32.0)	13 (9.0)	16 (14.0)	102 (67.5)	
Received adjuvant treatment, No. (%)					<.001
No treatment	73 (17.8)	71 (49.0)	2 (1.8)	0 (0.0)	
External beam radiotherapy	212 (51.7)	74 (51.0)	58 (50.9)	80 (53.0)	
Vaginal brachytherapy	54 (13.2)	0 (0.0)	54 (47.4)	0 (0.0)	
Chemoradiotherapy	71 (17.3)	0 (0.0)	0 (0.0)	71 (47.0)	

a *P* values reflect χ^2^ statistics or Fisher’s exact test for categorical variables and Kruskal-Wallis test for age. EC = endometrial cancer; EEC = endometrioid endometrial cancer; FIGO = International Federation of Gynecology and Obstetrics; IQR = interquartile range; MMRd = mismatch repair deficient; PORTEC = Post Operative Radiation Therapy in Endometrial Carcinoma.

### MMR Causes and Variant Analysis

**Figure 1.**
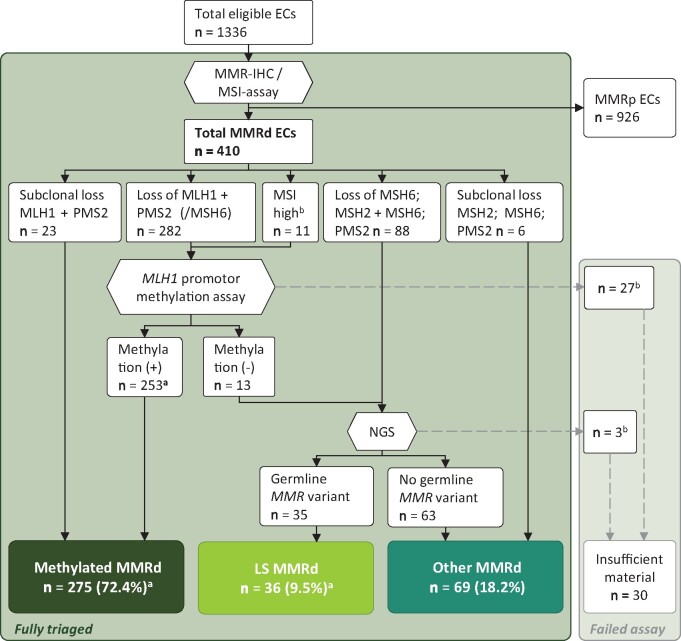
Flowchart. ^a^One case with *MLH1* promoter hypermethylation in the tumor carried a germline *MLH1* variant. ^b^Insufficient material for assay. EC = endometrial cancer; LS = Lynch syndrome; Methylation (+) = *MLH1* promoter hypermethylation; Methylation (−) = no *MLH1* promoter hypermethylation; MMR = mismatch repair; MMRd = mismatch repair deficient; MMRp = mismatch repair proficient; MSI = microsatellite instability; NGS = next-generation sequencing.

Complete triage was accomplished for 380 (92.7%) of the MMRd-ECs (Figure 1; insufficient material in 27 cases for *MLH1* methylation assay and 3 for NGS). Thirty-six *path_MMR* variant carriers were identified, giving a 2.8% LS prevalence in the overall population and a 9.5% LS prevalence within the MMRd group. There were 18 *path_MSH6*, 10 *path_PMS2*, 6 *path_MSH2*, and 2 *path_MLH1* variant carriers. An overview of the LS cases is displayed in Table 2. In total, 275 (72.4%) cases were classified as methylated. The remaining 69 (18.2%) MMRd cases were neither LS nor *MLH1* hypermethylated and were therefore classified as “other.”

**Table 2.** Patient and tumor characteristics of proven LS-associated ECs

No.	Study	Age, y	FIGO (2009)	Histotype	Grade	Molecular class by TCGA surrogate	Affected MMR proteins	MLH1 promoter methylation	Gene	Coding DNA mutation	Amino acid change	Class^a^
1	PORTEC-1	47	IB	Endometrioid	G2	MMRd	MSH2 + MSH6	NA	*MSH2*	c.1351C>T	p.(Gln451*)	5
2	PORTEC-1	67	IB	Endometrioid	G1	MMRd	MSH2 + MSH6	NA	*MSH2*	c.363T>G	p.(Tyr121*)	5
3	PORTEC-1	52	IA	Endometrioid	G3	MMRd	MSH2 + MSH6	NA	*MSH2*	c.646-2A>G	p.(?)	4
4	PORTEC-1	54	IA	Endometrioid	G1	MMRd	MSH2 + MSH6	NA	*MSH2*	c.2458 + 1G>A	p.(?)	4
5	PORTEC-3	48	IIIC	Endometrioid	G2	MMRd	MSH2 + MSH6	NA	*MSH2*	c.1285C>T	p.(Gln429*)	5
6	PORTEC-3	37	IIIC	Clear cell	G2	MMRd	MSH2 + MSH6	NA	*MSH2* ^b^	NA	NA	5
7	PORTEC-3	59	IA	Clear cell	G3	MMRd	MSH2 + MSH6	NA	*MSH6*	c.3188T>G	p.(leu1063Arg)	5
8	PORTEC-1	56	IA	Serous	G3	MMRd-p53abn	MSH2 subclonal + MSH6	NA	*MSH6*	c.1784delT	p.(Leu595Tyrfs*15)	5
9	PORTEC-1	67	IB	Endometrioid	G1	MMRd	MSH6	NA	*MSH6*	c.1189_1190insTT	p.(Tyr397Phefs*15)	5
10	PORTEC-1	58	IA	Endometrioid	G1	MMRd	MSH6	NA	*MSH6*	c.642C>A	p.(Tyr214*)	5
11	PORTEC-2	67	IB	Endometrioid	G1	MMRd	MSH6	NA	*MSH6*	c.2764C>T	p.(Arg922*)	5
12	PORTEC-2	66	IB	Endometrioid	G3	MMRd	MSH6	NA	*MSH6*	c.1483C>T	p.(Arg495*)	5
13	PORTEC-2	73	IB	Endometrioid	G1	MMRd-p53abn	MSH6	NA	*MSH6*	c.1628_1629delAA	p.(Lys543Argfs*19)	5
14	PORTEC-2	82	IIIA	Endometrioid	G1	MMRd	MSH6	NA	*MSH6*	c.3729_3732dupATTA	p.(Phe1245Ilefs*31)	5
15	PORTEC-2	71	IB	Endometrioid	G2	MMRd-p53abn	MSH6	NA	*MSH6*	c.2719_2720delGT	p.(Val907Argfs*10)	5
16	PORTEC-3	51	IIIA	Endometrioid	G1	MMRd	MSH6	NA	*MSH6*	c.3477C>A	p.(Tyr1159*)	5
17	PORTEC-3	55	IIIC	Endometrioid	G3	MMRd-p53abn	MSH6	NA	*MSH6*	c.2906_2907delAT	p.(Tyr969Leufs*5)	5
18	PORTEC-3	61	IB	Clear cell	G3	MMRd	MSH6	NA	*MSH6*	c.3838C>T	p.(Gln1280*)	5
19	PORTEC-3	68	IIIA	Endometrioid	G1	MMRd	MSH6	NA	*MSH6*	c.467C>G	p.(Ser156*)	5
20	PORTEC-3	59	IB	Serous	G3	MMRd-p53abn	MSH6	NA	*MSH6*	c.3527_3549delGACTTG GTGCCTCAGACAGAATA	p.(Arg1176Asnfs*4)	5
21	PORTEC-3	60	IA	Serous	G3	*POLE*mut-MMRd	MSH6	NA	*MSH6*	c.2342dupC	p.(Leu782Thrfs*3)	5
22	PORTEC-3	59	IB	Clear cell	G3	MMRd	MSH6	NA	*MSH6*	c.3863_3865dupAAT	p.(Phe1289*)	5
23	PORTEC-3	76	IB	Serous	G3	MMRd	MSH6	NA	*MSH6*	c.3847_3850dupATTA	p.(Thr1284Asnfs*6)	5
24	PORTEC-3	74	IA	Serous	G3	MMRd-p53abn	MSH6	NA	*MSH6*	c.10C>T	p.(Gln4*)	4
25	PORTEC-1	57	IB	Endometrioid	G3	MMRd	PMS2	NA	*PMS2*	c.1882C>T	p.(Arg628*)	5
26	PORTEC-1	66	IB	Endometrioid	G1	MMRd	PMS2	NA	*PMS2*	c.1882C>T	p.(Arg628*)	5
27	PORTEC-1	64	IB	Endometrioid	G3	MMRd-p53abn	PMS2	NA	*PMS2*	c.247_250dupTTAA	p.(Thr84Ilefs*9)	5
28	PORTEC-1	65	IB	Endometrioid	G1	MMRd	PMS2	NA	*PMS2*	c.1261C>T	p.(Arg421*)	5
29	PORTEC-2	61	IB	Endometrioid	G1	MMRd	PMS2	NA	*PMS2*	c.904_911delGTCTGCAG	p.(Val302Thrfs*4)	5
30	PORTEC-2	61	IB	Endometrioid	G3	MMRd	PMS2	NA	*PMS2*	c.1831dupA	p.(Ile611Asnfs*2)	5
31	PORTEC-2	78	IB	Endometrioid	G1	*POLE*mut-MMRd	PMS2	NA	*PMS2*	c.1882C>T	p.(Arg628*)	5
32	PORTEC-2	62	IB	Endometrioid	G2	MMRd	PMS2	NA	*PMS2*	c.904_911delGTCTGCAG	p.(Val302Thrfs*4)	5
33	PORTEC-3	54	IB	Endometrioid	G3	MMRd	PMS2	NA	*PMS2*	c.137G>T	p.(Ser46Ile)	5
34	PORTEC-3	48	II	Endometrioid	G3	MMRd	PMS2	NA	*PMS2*	c.989-2A>G	p.(Glu330_Glu381del)	4
35	PORTEC-3	52	IIIC	Endometrioid	G2	Not classified	MLH1 + PMS2	Methylated	*MLH1*	c.794G>C	p.(Arg265Pro)	4
36	PORTEC-1	48	IB	Endometrioid	G1	MMRd	MSI-high^c^	Unmethylated	MLH1	c.806C>G	p.(Ser269*)	5

a Classification according to the 5-tiered InSiGHT rules: class 5 is pathogenic and class 4 is likely pathogenic. G = grade; IHC = immunohistochemistry; LS = Lynch syndrome; MMRd = mismatch repair deficient; NA = not available; p53abn = p53 abnormal; POLEmut = POLE-ultramutated; PORTEC = Post Operative Radiation Therapy in Endometrial Carcinoma; TCGA = The Cancer Genome Atlas.

b Loss-of-function variant in *MSH2* gene identified by genetic testing (clinical data) but insufficient material for normal tissue next-generation sequencing.

c No material for MLH1 and PMS2 IHC.

**Figure 2.**
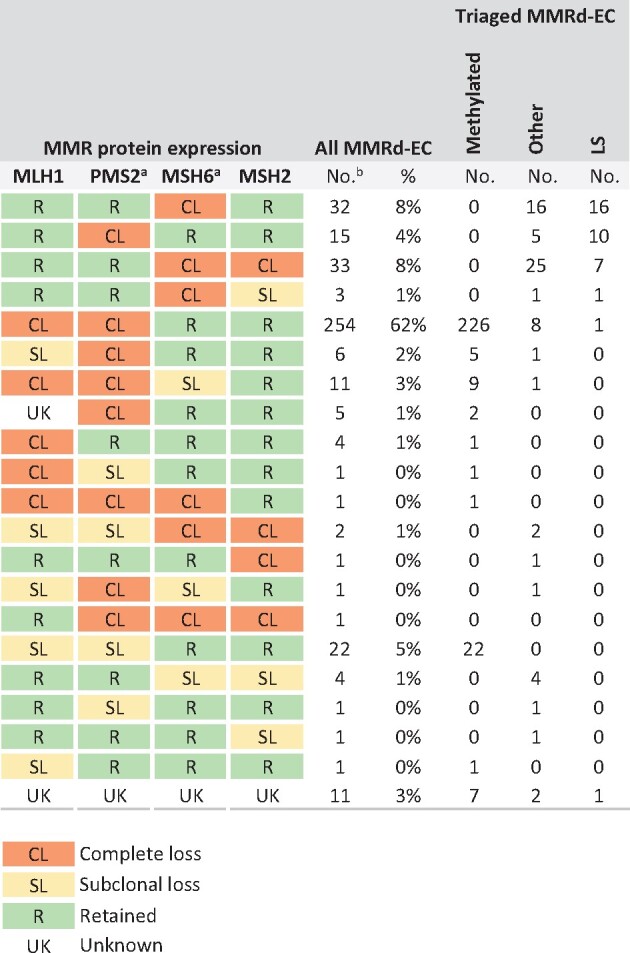
Details on the mismatch repair (MMR) protein expression according to the molecular cause of their MMR-deficient endometrial cancer (MMRd-EC). MMR protein expression was scored as following: complete loss (CL), retained (R), subclonal loss (SL), unknown/failed (UK). ^a^The concordance of these 2 columns shows that a 2-antibody (MSH6 and PMS2) panel is as sensitive as the full panel to detect Lynch syndrome (LS). ^b^All MMRd-ECs including those with insufficient material for *MLH1* methylation assay (n* *=* *27) and next-generation sequencing (n* *=* *3).

LS patients were younger, with a median age of 60 years (interquartile range = 54-67 years) and more often had p53 aberrant staining (20.0%) and serous (13.9%) or clear cell (8.3%) histology compared with the patients with methylated MMRd-EC (Table 3). Limiting screening of EC patients to age 50 years or younger, 60 years or younger, and 70 years or younger would have missed 31 (86.1%), 18 (50.0%), and 6 (16.7%) LS diagnoses, respectively. Figure 2 displays the distribution of the involved MMR proteins; all LS cases identified by the 4-panel approach would also have been identified by a 2-panel approach including only PMS2- and MSH6-IHC. No germline *POLE/POLD1* variants affecting function were identified. LS patients with *path_MSH6* and *path_PMS2* variants were older than those with *path_MLH1* and *path_MSH2* variants (median age = 63, 62, 50, and 50 years, respectively, *P *=* *.01; Supplementary Table 1, available online).

**Table 3.** Patient, tumor, and treatment characteristics according to the molecular cause of their MMRd-EC

Characteristic	All MMRd-EC	Methylated	Other	LS	*P* ^a^
Total, No. (%)	410^b^	275 (72.4)	69 (18.2)	36 (9.5)	
Age at random assignment					<.001
Median (IQR), y	65 (59-73)	67 (62-74)	59 (55-66)	60 (54-67)	
Trial, No. (%)					.002
PORTEC-1	145 (35.4)	99 (36.0)	22 (31.9)	12 (33.3)	
PORTEC-2	114 (27.8)	87 (31.6)	8 (11.6)	9 (25.0)	
PORTEC-3	151 (36.8)	89 (32.4)	39 (56.5)	15 (41.7)	
FIGO 2009 stage, No. (%)					.20
IA	104 (25.4)	70 (25.5)	17 (24.6)	7 (19.4)	
IB	200 (48.8)	137 (49.8)	27 (39.1)	21 (58.3)	
II	36 (8.8)	22 (8.0)	11 (15.9)	1 (2.8)	
III	70 (17.1)	46 (16.7)	14 (20.3)	7 (19.4)	
Histological grade and type, No. (%)					<.001
EEC grade 1 or 2	274 (66.8)	197 (71.6)	40 (58.0)	19 (52.8)	
EEC grade 3	99 (24.1)	64 (23.3)	18 (26.1)	8 (22.2)	
Serous	11 (2.7)	2 (0.7)	4 (5.8)	5 (13.9)	
Clear cell	12 (2.9)	2 (0.7)	6 (8.7)	3 (8.3)	
Other	14 (3.4)	10 (3.6)	1 (1.4)	1 (2.8)	
Myometrial invasion, No. (%)					.41
>50%	274 (66.8)	187 (68.0)	43 (62.3)	27 (75.0)	
Lymphovascular space invasion, No. (%)					.96
Present	131 (32.0)	90 (32.7)	23 (33.3)	11 (30.6)	
*POLE*mut in tumor, No. (%)					.002
Exonuclease domain mutations	19 (4.7)	8 (2.9)	9 (13.4)	2 (5.7)	
p53 immunohistochemistry, No. (%)					<.001
Aberrant	31 (7.7)	7 (2.6)	14 (20.9)	7 (20.0)	
Received adjuvant treatment, No. (%)					.10
No treatment	73 (17.8)	47 (17.1)	10 (14.5)	9 (25.0)	
External beam radiotherapy	212 (51.7)	145 (52.7)	40 (58.0)	13 (36.1)	
Vaginal brachytherapy	54 (13.2)	39 (14.2)	3 (4.3)	6 (16.7)	
Chemoradiotherapy	71 (17.3)	44 (16.0)	16 (23.2)	8 (22.2)	

^a^ *P* values reflect χ^2^ statistics or Fisher’s exact test for categorical variables and Kruskal-Wallis test for age. EC = endometrial cancer; EEC = endometrioid endometrial cancer; FIGO = International Federation of Gynecology and Obstetrics; IQR = interquartile range; MMRd = mismatch repair deficient; *POLE*mut = *POLE*-ultramutated; PORTEC = Post Operative Radiation Therapy in Endometrial Carcinoma.

^b^ All MMRd-ECs including those with insufficient material for *MLH1*-methylation assay (n* *=* *27) and normal tissue next-generation sequencing (n* *=* *3).

### Survival

**Figure 3.**
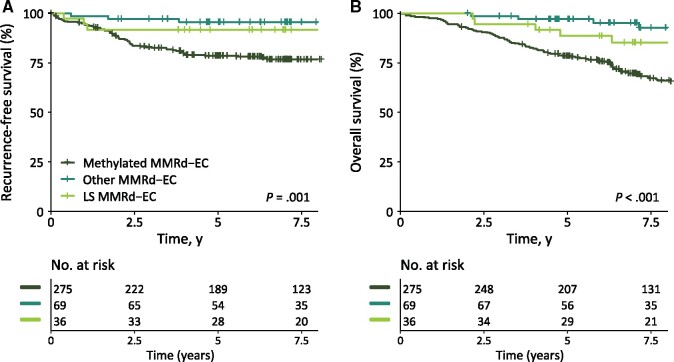
Kaplan-Meier survival curves for recurrence-free survival (A) and overall survival (B) for patients with methylated mismatch repair deficient (MMRd), other MMRd and Lynch syndrome (LS) associated MMRd endometrial cancer (EC). All cases with MMRd phenotype are included in this analysis, including cases with a concurrent *POLE* variant affecting function (*POLE*mut-MMRd-EC). *P* values reflect 2-sided log-rank test.

The estimated RFS for the MMRd population at 5 years was 83.7% (95% CI = 80.1% to 87.4%): 91.7% (95% CI = 83.1% to 100%) for patients with LS-associated MMRd-EC, 78.6% (95% CI = 73.8% to 83.7%) for patients with methylated MMRd-EC, and 95.5% (95% CI = 90.7% to 100%) for patients with other causes of MMRd-EC (*P *=* *.001; Figure 3, A; LS vs methylated: HR = 0.45, 95% CI = 0.16 to 1.24, *P *=* *.12; other vs methylated: HR = 0.17, 95% CI = 0.05 to 0.55, *P *=* *.003).

The estimated OS for the MMRd population at 5 years was 82.8% (95% CI = 79.2% to 86.5%): 88.5% (95% CI = 78.5% to 99.8%) for patients with LS-associated MMRd-EC, 78.5% (95% CI = 73.7% to 83.5%) for patients with methylated MMRd-EC, and 97.0% (95% CI = 93.0% to 100%) for patients with other causes of MMRd-EC (*P *<* *.001; Figure 3, B; LS vs methylated: HR = 0.50, 95% CI = 0.24 to 1.02, *P *=* *.06; other vs methylated: HR = 0.27, 95% CI = 0.13 to 0.55, *P *<* *.001). After adjustment for age, the trend for better OS in the LS group was no longer observed (vs methylated MMRd-EC: HR = 0.73, 95% CI = 0.35 to 1.52, *P *=* *.40), whereas age and having another cause of MMRd were statistically significant prognostic factors (HR = 1.07, 95% CI = 1.04 to 1.09, *P *<* *.001; other vs methylated MMRd-EC: HR = 0.41, 95% CI = 0.20 to 0.85, *P *=* *.02).

### Second Primary Cancers

At 10 years, the cumulative incidence of developing a second LS-associated tumor was 11.6% (95% CI = 0.0% to 24.7%) among EC patients with LS, 1.5% (95% CI = 0.0% to 4.3%) among patients with other MMRd-EC, and 7.0% (95% CI = 3.0% to 10.9%) among patients with methylated MMRd-EC (Supplementary Figure 2, available online). Three of the 4 LS-patients who developed a second primary LS-associated cancer had colon cancer (after 3.8, 4.8, and 14.9 years) and 1 had ureteral cancer (after 8.0 years; Supplementary Table 2, available online, shows cancer type distribution). The cause-specific hazard ratio for developing an LS-associated second cancer was 1.9 (95% CI = 0.63 to 5.7, *P *=* *.26) for patients with LS vs patients with methylated MMRd-EC.

## Discussion

After complete IHC-based tumor triage, we found a 2.8% prevalence of LS in 1 of the largest EC trial populations worldwide, comprising all risk groups with long and complete follow-up. The prevalence of LS among patients with MMRd-EC was 9.5%. Patients with LS were relatively young, but restricted testing to women who are 60 years or younger would have missed one-half of the cases. Patients with LS tend to have a better RFS and a higher risk of developing second primary cancers compared with patients with methylated MMRd-ECs. No trend for more favorable OS was found after adjustment for age.

This is the first study to our knowledge investigating the prognostic value of LS within the MMRd-EC subgroup. Most of the recent research showed that MMRd-ECs, predominantly driven by the large number of *MLH1* hypermethylated cases, have an intermediate prognosis within the molecular classification introduced by The Cancer Genome Atlas (10‐12). Our survival analysis showed that EC patients with LS tend to have a better RFS than patients with methylated MMRd-EC (HR = 0.45, *P *=* *.12), whereas LS had no statistically significant prognostic value for OS after adjustment for age (age-adjusted HR = 0.73, *P *=* *.40). The favorable prognosis has been assumed to be induced by the active local immune response (14,27). Comparable survival analysis in CRC has been published. One study showing a better OS for 85 CRC patients with LS compared with 67 sporadic MMRd patients after adjustment for age, stage, and *BRAF* status (HR = 0.29, 95% CI = 0.09 to 0.95, *P *=* *.04) (28). The other study also showing better OS in 37 CRC patients with LS compared with 106 methylated MMRd patients, although the difference was minimal after adjusting for age and stage (29).

The cumulative incidence for developing a second LS-associated cancer at 10 years was 11.6% (95% CI = 0.0% to 24.7%) for patients with LS vs 7.0% (95% CI = 3.0% to 10.9%) for patients with methylated MMRd-EC (HR = 1.90, 95% CI = 0.63 to 5.7, *P *=* *.26). Our analysis was underpowered due to the small number of events in the LS group. Nevertheless, the elevated risk strengthens previous reports on subsequent cancers in EC or non-CRC LS patients (15%-24%) (4,30) and is of importance for surveillance strategies.

The 2.8% prevalence of LS-EC is consistent with previous publications in which prevalences of 2.8%-3.2% were reported (15‐17). This prevalence is likely a slight underestimation. Firstly, our NGS panel did not include *EPCAM* and could not detect large rearrangements. To detect large rearrangement in *EPCAM* or the MMR genes, Multiplex Ligation-dependent Probe Amplification is most commonly used but performs poorly on FFPE tissue. Secondly, the patient selection in our trial design may have affected the prevalence. Patients younger than 60 years with stage I ECs were excluded from the PORTEC-2 trial. Nevertheless, the total PORTEC population deviates minimally from the general EC population as suggested by the similar age in the Proportion of Endometrial Tumours Associated Lynch Syndrome study, an unselected, prospective, cross-sectional study in the United Kingdom among 500 EC patients (15). Moreover, patients with a history of cancer were excluded from the PORTEC trials. The PORTEC population represents women with EC as their sentinel LS-associated malignancy, which is the case in more than one-half of those women with LS who develop cancer (3). Although this selection has potentially led to a slight underestimation of the prevalence of LS in EC, it does represent the patients in which LS could be detected by IHC-based tumor triage. The recently published meta-analysis by Ryan et al. (16) included mostly small trials with methodological heterogeneity, often selecting their test population by age and/or family history, and incomplete testing; only 1 publication included over 1000 ECs, but germline testing was limited to the minority of the triaged potential LS cases (6). Our study is the first with LS testing in an EC population consisting of more than 1000 women with almost complete MMR-IHC, targeted *MLH1* methylation testing, and MMR germline testing, making our estimates more reliable.

The *path_MSH6* carrier rate of 50.0% among the PORTEC patients with LS is consistent with LS testing results in other unselected EC populations (15,17), but it is remarkably high compared with LS registry data. Only 13% of *path_MMR* carriers in the clinically selected Prospective Lynch Syndrome Database bear *path_MSH6* [1]. As mentioned above, our cohort represents patients with EC as their sentinel cancer likely to induce a lower frequency of *path_MLH1* and *path_MSH2*. Moreover, it must be considered that most of our participants were Dutch, and the *path_MSH6* rate of 30% among the Dutch LS registry patients was relatively high compared with the overall Prospective Lynch Syndrome Database (31). Lastly, *path_MSH6* families are not identified efficiently by current clinical criteria for LS (32) due to the later age of onset of CRC, incomplete penetrance, and a higher risk and later age of onset of EC (1,33‐35). The same applies to *path_PMS2* carriers with a substantially lower cancer risk (1,2,15,16). Correspondingly, the *path_MSH6* and *path_PMS2* carriers were older than *the path_MLH1* and *path_MSH2* carriers in our population.

Triage of incident ECs based on IHC with targeted *MLH1* methylation testing, as has been adopted widely for CRC, may be a more effective strategy to identify these LS families than age- and family history–based triage. An upper age screening limit would not be recommended, because limiting screening to EC patients who are aged 70 years or younger would have missed 6 (16.7%) LS diagnoses. We confirmed that a 2-antibody panel including MSH6- and PMS2-IHC, with MSH2- or MLH1-IHC only in case of inconclusive staining, is as sensitive as the full panel to detect LS, so this could be a reliable alternative to improve cost-effectiveness (5,36).

A limitation of our study was the lack of germline LS sequencing on the whole study population. Therefore, sensitivity of the IHC-based triage to identify LS patients could not be assessed. Some patients with LS might have been diagnosed before entering the trial, although many were diagnosed after inclusion and had no prior knowledge of the germline mutation.

The diagnosis of LS in EC is crucial for counseling and cancer surveillance even though these patients might be older than those presenting with CRC (18). Moreover, LS screening in incident ECs will have consequences for the patient’s family. Cascade testing of at-risk relatives can identify *path_MMR* carriers who can benefit from cancer surveillance and risk-reducing treatment (37,38). The clinical impact depends on the gene-specific cancer risk and is substantially lower for *path_PMS2* carriers (1,2). Finally, LS identification may have consequences by allowing clinicians to better estimate and explain prognosis and to potentially tailor treatment in the upcoming immunotherapy era (14,27,39).

Further research into the causes of the 63 cases with neither *MLH1* hypermethylation nor a *MMR* germline variant is ongoing. It is hypothesized that the majority will be explained by a sporadic origin through biallelic somatic MMR inactivation (15,40). The determination of a sporadic explanation excludes potential undetectable LS (or Lynch-like syndrome) and will avoid a clinical management dilemma in those cases.

In conclusion, LS was identified using MMR-IHC with targeted *MLH1* methylation–based triage in 2.8% of 1336 patients with EC from the combined PORTEC-1, -2, and -3 trials, corresponding to 9.5% of the MMRd tumors. LS was mainly caused by germline variants in the *MSH6* and *PMS2* genes. Patients with LS-associated ECs showed a trend towards better RFS and higher risk for second primary cancers compared with patients with ECs caused by *MLH1* hypermethylation. Besides a prognostic impact, screening all incident ECs without an upper age limit to identify LS using tumor-based triage may benefit counseling, affect treatment decisions, and facilitate prevention strategies for current and future patients and their families.

## Funding

This study was supported by the Dutch Cancer Society (UL2012-5719). Translational research was performed on tissue samples from 3 randomized clinical trials (PORTEC-1, PORTEC-2, and PORTEC-3). The trials were supported by grants from the Dutch Cancer Society (CKTO 90–01, CKTO 2001–04, and UL2006-4168/CKTO 2006–04).

## Notes

**Role of the funder:** The Dutch Cancer society had no role in the design and conduct of the study; collection, managements, analysis, and interpretation of the data; or decision to submit the manuscript for publication.

**Disclosures:** CLC reports grants from Dutch Cancer Society. TB reports grants from Dutch Cancer Society. RN reports grants from Elekta, Varian and Accuray, outside the submitted work. EJC was supported by the National Institute for Health Research Manchester Biomedical Research Centre (IS-BRC-1215–20007). All remaining authors have declared no conflicts of interest.

**Author contributions:** Conceptualization, CCBP, ES, VTHBMS, RAN, TB, CLC; Methodology, CCBP, ES, RAN, HP, NH, TvW, TB, CLC. Validation, CMT, TvW. Formal Analysis, CCBP, HP, NH; Investigation, CCBP, ES, DR, TAR, TvW. Resources, IMJS, LCHWL, JJJ, RAN, EJC, MEP, LM, AL, CCR. Data curation, CCBP, LV, SMdB, NH; Writing—Orginal Draft, CCBP, TB, CLC; Writing—Review & Editing, CCBP, ES, VTHBMS, DR, CMT, LV, TAR, IMJS, LCHWL, JJJ, RAN, EJC, MEP, LM, AL, PB, HP, SMdB, NH, MN, TvW, TB, CLC. Visualization, CCBP; Supervision, TB, CLC. Funding Acquisition, TB, CLC.

**Prior presentation:** The study was presented in part at 2020 International Gynecologic Cancer Society xDigital Annual Global Meeting (September 10-12, 2020).

**Acknowledgements:** We thank all clinical and pathology teams at participating sites of the PORTEC and TransPORTEC study groups, as well as the women who participated in the trials and their families. We thank Alicia León-Castillo, Natalja T. ter Haar, Michelle Osse and Enno J. Dreef (Leiden University Medical Center) for excellent technical support, and Lisette M. Wiltink (Leiden University Medical Center) for verification of the second cancers in PORTEC-1 and -2 trials in the Dutch Pathology Registry (PALGA). We also thank Karen Verhoeven-Adema (Comprehensive Cancer Center, the Netherlands) for her dedicated work as central PORTEC-3 data manager and trial coordinator.

## Data Availability

The data underlying this article will be shared on reasonable request to the corresponding author.

## Supplementary Material

djab029_Supplementary_Data

## References

[1] Dominguez-Valentin M, Sampson JR, Seppala TT, et al Cancer risks by gene, age, and gender in 6350 carriers of pathogenic mismatch repair variants: findings from the Prospective Lynch Syndrome Database. Genet Med. 2020;22(1):15–25. 31337882 10.1038/s41436-019-0596-9 PMC7371626

[2] Ten Broeke SW, van der Klift HM, Tops CMJ, et al Cancer risks for PMS2-associated Lynch syndrome. J Clin Oncol. 2018;36(29):2961–2968. 30161022 10.1200/JCO.2018.78.4777 PMC6349460

[3] Lu KH, Dinh M, Kohlmann W, et al Gynecologic cancer as a “sentinel cancer” for women with hereditary nonpolyposis colorectal cancer syndrome. Obstet Gynecol. 2005;105(3):569–574. 15738026 10.1097/01.AOG.0000154885.44002.ae

[4] Win AK, Lindor NM, Winship I, et al Risks of colorectal and other cancers after endometrial cancer for women with Lynch syndrome. J Natl Cancer Inst. 2013;105(4):274–279. 23385444 10.1093/jnci/djs525 PMC3576323

[5] Stelloo E, Jansen AML, Osse EM, et al Practical guidance for mismatch repair-deficiency testing in endometrial cancer. Ann Oncol. 2016;28(1):96–102. 10.1093/annonc/mdw542 27742654

[6] Goodfellow PJ, Billingsley CC, Lankes HA, et al Combined microsatellite instability, MLH1 methylation analysis, and immunohistochemistry for Lynch syndrome screening in endometrial cancers from GOG210: an NRG Oncology and Gynecologic Oncology Group Study. J Clin Oncol. 2015;33(36):4301–4308. 26552419 10.1200/JCO.2015.63.9518 PMC4678181

[7] Geurts-Giele WR, Leenen CH, Dubbink HJ, et al Somatic aberrations of mismatch repair genes as a cause of microsatellite-unstable cancers. J Pathol. 2014;234(4):548–559. 25111426 10.1002/path.4419

[8] Mensenkamp AR, Vogelaar IP, van Zelst-Stams WA, et al Somatic mutations in MLH1 and MSH2 are a frequent cause of mismatch-repair deficiency in Lynch syndrome-like tumors. Gastroenterology. 2014;146(3):643–646. e8. 24333619 10.1053/j.gastro.2013.12.002

[9] Haraldsdottir S, Hampel H, Tomsic J, et al Colon and endometrial cancers with mismatch repair deficiency can arise from somatic, rather than germline, mutations. Gastroenterology. 2014;147(6):1308–1316.e1. 25194673 10.1053/j.gastro.2014.08.041 PMC4294551

[10] Cancer Genome Atlas Research Network. Integrated genomic characterization of endometrial carcinoma. Nature. 2013;497(7447):67–73. 23636398 10.1038/nature12113 PMC3704730

[11] Stelloo E, Nout RA, Osse EM, et al Improved risk assessment by integrating molecular and clinicopathological factors in early-stage endometrial cancer-combined analysis of the PORTEC cohorts. Clin Cancer Res. 2016;22(16):4215–4224. 27006490 10.1158/1078-0432.CCR-15-2878

[12] Leon-Castillo A, de Boer SM, Powell ME, et al; on behalf of the TransPORTEC consortium. Molecular classification of the PORTEC-3 trial for high-risk endometrial cancer: impact on prognosis and benefit from adjuvant therapy. J Clin Oncol. 2020;38(29):3388–3397. 32749941 10.1200/JCO.20.00549 PMC7527156

[13] Ott PA, Bang YJ, Piha-Paul SA, et al T-cell-inflamed gene-expression profile, programmed death ligand 1 expression, and tumor mutational burden predict efficacy in patients treated with pembrolizumab across 20 cancers: KEYNOTE-028. J Clin Oncol. 2019;37(4):318–327. 30557521 10.1200/JCO.2018.78.2276

[14] Ramchander NC, Ryan NAJ, Walker TDJ, et al Distinct immunological landscapes characterize inherited and sporadic mismatch repair deficient endometrial cancer. Front Immunol. 2019;10:3023. 31998307 10.3389/fimmu.2019.03023 PMC6970202

[15] Ryan NAJ, McMahon R, Tobi S, et al The proportion of endometrial tumours associated with Lynch syndrome (PETALS): a prospective cross-sectional study. PLoS Med. 2020;17(9):e1003263. 32941469 10.1371/journal.pmed.1003263 PMC7497985

[16] Ryan NAJ, Glaire MA, Blake D, et al The proportion of endometrial cancers associated with Lynch syndrome: a systematic review of the literature and meta-analysis. Genet Med. 2019;21(10):2167–2180. 31086306 10.1038/s41436-019-0536-8 PMC8076013

[17] Hampel H, Pearlman R, de la Chapelle A, et al Double somatic mismatch repair gene pathogenic variants as common as Lynch syndrome among endometrial cancer patients. Gynecol Oncol. 2021;160(1):161–168. 33393477 10.1016/j.ygyno.2020.10.012 PMC7783191

[18] Hampel H, Frankel WL, Martin E, et al Screening for the Lynch syndrome (hereditary nonpolyposis colorectal cancer). N Engl J Med. 2005;352(18):1851–1860. 15872200 10.1056/NEJMoa043146

[19] Nout RA, Poll-Franse LVvd, Lybeert MLM, et al Long-term outcome and quality of life of patients with endometrial carcinoma treated with or without pelvic radiotherapy in the Post Operative Radiation Therapy in Endometrial Carcinoma 1 (PORTEC-1) trial. J Clin Oncol. 2011;29(13):1692–1700. 21444867 10.1200/JCO.2010.32.4590

[20] Wortman BG, Creutzberg CL, Putter H, et al Ten-year results of the PORTEC-2 trial for high-intermediate risk endometrial carcinoma: improving patient selection for adjuvant therapy. Br J Cancer. 2018;119(9):1067–1074. 30356126 10.1038/s41416-018-0310-8 PMC6219495

[21] de Boer SM, Powell ME, Mileshkin L, et al Adjuvant chemoradiotherapy versus radiotherapy alone in women with high-risk endometrial cancer (PORTEC-3): patterns of recurrence and post-hoc survival analysis of a randomised phase 3 trial. Lancet Oncol. 2019;20(9):1273–1285. 31345626 10.1016/S1470-2045(19)30395-X PMC6722042

[22] Leon-Castillo A, Gilvazquez E, Nout R, et al Clinicopathological and molecular characterisation of ‘multiple-classifier’ endometrial carcinomas. J Pathol. 2020;250(3):312–322. 31829447 10.1002/path.5373 PMC7065184

[23] Deng G, Chen A, Hong J, et al Methylation of CpG in a small region of the hMLH1 promoter invariably correlates with the absence of gene expression. Cancer Res. 1999;59(9):2029–2033. 10232580

[24] Jansen AML, Tops CMJ, Ruano D, et al The complexity of screening PMS2 in DNA isolated from formalin-fixed paraffin-embedded material. Eur J Hum Genet. 2020;28(3):333–338. 31616036 10.1038/s41431-019-0527-x PMC7028990

[25] Cohen D, Hondelink LM, Solleveld-Westerink N, et al Optimizing mutation and fusion detection in NSCLC by Sequential DNA and RNA sequencing. J Thorac Oncol. 2020;15(6):1000–1014. 32014610 10.1016/j.jtho.2020.01.019

[26] Daniel WW. Biostatistics: A Foundation for Analysis in the Health Sciences. 7th ed. New York: John Wiley & Sons; 1999.

[27] Pakish JB, Zhang Q, Chen Z, et al Immune microenvironment in microsatellite-instable endometrial cancers: hereditary or sporadic origin matters. Clin Cancer Res. 2017;23(15):4473–4481. 28264871 10.1158/1078-0432.CCR-16-2655 PMC5540763

[28] Liu GC, Liu RY, Yan JP, et al The heterogeneity between lynch-associated and sporadic MMR deficiency in colorectal cancers. J Natl Cancer Inst. 2018;110(9):975–984. 29471527 10.1093/jnci/djy004

[29] Haraldsdottir S, Hampel H, Wu C, et al Patients with colorectal cancer associated with Lynch syndrome and MLH1 promoter hypermethylation have similar prognoses. Genet Med. 2016;18(9):863–868. 26866578 10.1038/gim.2015.184 PMC5489337

[30] Moller P, Seppala T, Bernstein I, et al; Mallorca Group (http://mallorca-group.org). Incidence of and survival after subsequent cancers in carriers of pathogenic MMR variants with previous cancer: a report from the prospective Lynch syndrome database. Gut. 2017;66(9):1657–1664. 27261338 10.1136/gutjnl-2016-311403 PMC5561364

[31] Woolderink JM, De Bock GH, de Hullu JA, et al Characteristics of Lynch syndrome associated ovarian cancer. Gynecol Oncol. 2018;150(2):324–330. 29880284 10.1016/j.ygyno.2018.03.060

[32] Sjursen W, Haukanes BI, Grindedal EM, et al Current clinical criteria for Lynch syndrome are not sensitive enough to identify MSH6 mutation carriers. J Med Genet. 2010;47(9):579–585. 20587412 10.1136/jmg.2010.077677 PMC2976029

[33] LaDuca H, Polley EC, Yussuf A, et al A clinical guide to hereditary cancer panel testing: evaluation of gene-specific cancer associations and sensitivity of genetic testing criteria in a cohort of 165,000 high-risk patients. Genet Med. 2020;22(2):407–415. 31406321 10.1038/s41436-019-0633-8 PMC7000322

[34] Ryan NAJ, Morris J, Green K, et al Association of mismatch repair mutation with age at cancer onset in lynch syndrome: implications for stratified surveillance strategies. JAMA Oncol. 2017;3(12):1702–1706. 28772289 10.1001/jamaoncol.2017.0619 PMC5824283

[35] Bonadona V, Bonaiti B, Olschwang S, et al; French Cancer Genetics Network. Cancer risks associated with germline mutations in MLH1, MSH2, and MSH6 genes in Lynch syndrome. Jama. 2011;305(22):2304–2310. 21642682 10.1001/jama.2011.743

[36] Snowsill TM, Ryan NAJ, Crosbie EJ. Cost-effectiveness of the Manchester approach to identifying Lynch syndrome in women with endometrial cancer. J Clin Med. 2020;9(6):1664. 10.3390/jcm9061664 PMC7356917 32492863

[37] Burn J, Sheth H, Elliott F, et al Cancer prevention with aspirin in hereditary colorectal cancer (Lynch syndrome), 10-year follow-up and registry-based 20-year data in the CAPP2 study: a double-blind, randomised, placebo-controlled trial. Lancet. 2020;395(10240):1855–1863. 32534647 10.1016/S0140-6736(20)30366-4 PMC7294238

[38] Moller P, Seppala T, Bernstein I, et al; Mallorca Group (http://mallorca-group.eu). Cancer incidence and survival in Lynch syndrome patients receiving colonoscopic and gynaecological surveillance: first report from the prospective Lynch syndrome database. Gut. 2017;66(3):464–472. 26657901 10.1136/gutjnl-2015-309675 PMC5534760

[39] Horeweg N, de Bruyn M, Nout RA, et al Prognostic integrated image-based immune and molecular profiling in early-stage endometrial cancer. Cancer Immunol Res. 2020;8(12):1508–1519. 32999003 10.1158/2326-6066.CIR-20-0149

[40] Buchanan DD, Clendenning M, Jayasekara H, et al Double somatic mutations as a cause of tumor mismatch repair-deficiency in population-based colorectal and endometrial cancer with Lynch-like syndrome. Cancer Res. 2017;77(13 Suppl):Abstract nr 4266.

