# Supplementary material for: Prevalence and Prognosis of Lynch Syndrome and Sporadic Mismatch Repair Deficiency in Endometrial Cancer
Source: J Natl Cancer Inst. 2021 Mar 8;113(9):1212–20. doi: 10.1093/jnci/djab029 (PMC8418420; doi:10.1093/jnci/djab029)
Supplement: djab029_Supplementary_Data [file djab029_supplementary_data.pdf]

SUPPLEMENTARY MATERIALS

Supplementary Methods

<u>Definitions</u>

MMRd-EC = All EC with loss of expression of one or more MMR proteins and positive internal control irrespective of POLE- and MSI-status. In case MSI testing was performed, but no MMR-IHC, all EC with MSI-high status. In case not all four MMR proteins could be stained: All EC with loss of expression of at least one MMR protein or MSI-high status.

MMRp-EC = All EC with retained expression of all four MMR proteins. In case MSI testing is performed, but no MMR-IHC, all EC with MSI-low or MSS status.

Suspected of Lynch syndrome/Potential LS-associated MMRd-EC = All MMRd-EC with loss of MLH1 expression without hypermethylation of the *MLH1* promotor; loss of MSH2 and/or MSH6 expression or isolated loss of PMS2 expression. In case MSI testing was performed, but no MMR-IHC, all MMRd-EC with MSI-high status without hypermethylation of the *MLH1* promotor. In case not all four MMR proteins could be stained: MMRd-EC with loss of MSH2, MSH6 and/or PMS2 with retained MLH1 expression, or loss of MLH1 expression or MSI-high if MLH1-IHC is not available without *MLH1* promotor hypermethylation.

LS-associated MMRd-EC = MMRd-EC with a germline variant (likely) affecting function corresponding with MMR protein loss. Class 4 or 5 according to InSiGHT Variant Classification.

MMRd caused by *MLH1* promotor hypermethylation = All MMRd-EC with loss of MLH1 expression and proven *MLH1* promotor hypermethylation by methylation specific PCR. In case MSI testing was performed, but no MMR-IHC, all EC with MSI-high status and proven *MLH1* promotor hypermethylation. In case not all four MMR proteins could be stained: MMRd-EC with loss of PMS2 and/or MLH1 expression and proven *MLH1* promotor hypermethylation. Also including cases with subclonal loss of MLH1 and total loss of PMS2 expression with *MLH1* promotor hypermethylation.

Subclonal loss of MMR expression = Subclonal loss (≥10%) of one or more MMR proteins (NB excluding cases with complete loss of expression of another MMR protein, than the complete loss of another MMR protein is leading in group allocation).

Methylated MMRd-EC = All EC with MMRd caused by *MLH1* promotor hypermethylation and subclonal loss of MLH1 expression.

MMRd-EC with other causes = MMRd-EC with neither a MMR germline variant affecting function in DNA isolated from normal tissue nor promotor hypermethylation of *MLH1* in the tumor. A mixed group having alternative causes of MMRd. It is hypothesised that the majority will be explained by sporadic origin through biallelic somatic MMR inactivation (i.e. variants affecting function or loss of heterozygosity [LOH]), and few cases may have an undetectable hereditary syndrome (frequently referred to as ‘Lynch-like syndrome’ in literature).

MMRd-EC with unknown *MLH1* methylation status = All MMRd-EC with loss of MLH1 expression and insufficient material for *MLH1* promoter methylation assay.

Complete triage/Fully triaged = All identified MMRd-EC with successful *MLH1* promoter methylation assay and next-generation sequencing when indicated.

Definition		Depending on performed MMR/MSI test(s)			MLH1 methylation status	NGS
		**MMR-IHC + MSI test or only MMR-IHC**	**Only MSI-test**	**MMR-IHC of <4 proteins performed + MSI-test**	**-**	**-**
MMRd		Loss of ≥1 MMR proteins	MSI-high	Loss of ≥1 MMR proteins or MSI high	-	-
MMRp		All retained expression	MSI low or MSS	all IHC retained expression +MSI low or MSS	-	-
Suspected of LS / Potential LS-associated		MSH2 and/or MSH6 loss, PMS2 loss		MSH2, MSH6 or PMS2 loss with retained MLH1	-	-
		MLH1 loss	MSI-high	MLH1 loss or MSI-high if MLH1-IHC N/A	Unmethylated	-
Methylated MMRd-EC<br><br>Included subgroups:<br>1. *MLH1* methylated;<br>2. Subclonal MLH1 loss;	1	MLH1 loss	MSI-high	PMS2 and/or MLH1 loss or MSI-high if MLH1-IHC N/A	Hypermethylated	-
	2	Subclonal MLH1 loss[a]	-		-	-
LS-associated EC		Suspected of LS; Corresponding to NGS	-	Suspected of LS; Corresponding to NGS	-	Pathogenic mutation in normal (and tumor) tissue
MMRd-EC with other cause<br><br>Included subgroups:<br>1. Explained somatic<br>2 (a/b). Unexplained	1	Suspected of LS or Subclonal MSH2, MSH6 or PMS2 loss[a]	Suspected of LS	Suspected of LS	-	Double somatic mutations in tumor without pathogenic mutation in normal tissue (Ongoing research)
	2a				-	No pathogenic mutation in normal tissue found and no double somatic alteration in tumor (Ongoing research)
	2b				-	No pathogenic mutation in normal tissue found and tumor NGS failed
Failed cases<br>Included subgroups:<br>1. Unknown *MLH1* methylation status<br>2. Suspected of LS but failed NGS	1	MLH1 loss		PMS2 and/or MLH1 loss	Failed	-
	2	Suspected of LS	Suspected of LS	Suspected of LS	-	Normal tissue NGS failed

Supplementary Figure 1. Definitions

The definition depend on the combination of the results of the MMR/MSI test (choose one of the three green columns based on the available test results; MMR-IHC [dark green column] is preferable when available), *MLH1* methylation status (blue) and NGS (orange) in the corresponding row.

[a] NB excluding cases with complete loss of another MMR protein; the complete loss of another MMR protein is leading in group allocation.

EC= endometrial cancer; IHC = immunhistochemistry; LS = Lynch syndrome; MMRd = mismatch repair deficient; MMRp = mismatch repair proficient; MSI = microsatellite instability; N/A = not available; NGS = next-generation sequencing

Supplementary Table 1. Patient, tumor and treatment characteristics of patients with proven MMR germline variant according to affected gene

	MLH1<br>*n*=2	PMS2<br>*n*=10	MSH2<br>*n*=6	MSH6<br>*n*=18	*p*-value
**Age at randomization**					**0.01**
Median (IQR), y	50 (49-51)	62 (58-65)	50 (47-54)	63 (59-70)	
**Trial, No. (%)**					0.18
PORTEC-1	1 (50.0)	4 (40.0)	4 (66.7)	3 (16.7)	
PORTEC-2	0 (0.0)	4 (40.0)	0 (0.0)	5 (27.8)	
PORTEC-3	1 (50.0)	2 (20.0)	2 (33.3)	10 (55.6)	
**FIGO 2009 stage, No. (%)**					0.20
IA	0 (0.0)	0 (0.0)	2 (33.3)	5 (27.8)	
IB	1 (50.0)	9 (90.0)	2 (33.3)	9 (50.0)	
II	0 (0.0)	1 (10.0)	0 (0.0)	0 (0.0)	
III	1 (50.0)	0 (0.0)	2 (33.3)	4 (22.2)	
**Histological grade and type, No. (%)**					0.30
EEC grade 1/2	2 (100.0)	5 (50.0)	4 (66.7)	8 (44.4)	
EEC grade 3	0 (0.0)	5 (50.0)	1 (16.7)	2 (11.1)	
Serous	0 (0.0)	0 (0.0)	0 (0.0)	5 (27.8)	
Clear cell	0 (0.0)	0 (0.0)	1 (16.7)	2 (11.1)	
Other	0 (0.0)	0 (0.0)	0 (0.0)	1 (5.6)	
**Myometrial invasion, No. (%)**					0.18
>50%	1 (50.0)	10 (100.0)	4 (66.7)	12 (66.7)	
**LVSI, No. (%)**					0.57
Present	0 (0.0)	2 (20.0)	2 (33.3)	7 (38.9)	
***POLE*mut in tumour , No. (%)**					0.86
EDM	0 (0.0)	1 (10.0)	0 (0.0)	1 (5.6)	
**p53 IHC, No. (%)**					0.22
Aberant	0 (0.0)	1 (10.0)	0 (0.0)	6 (33.3)	
**Received Adjuvant Treatment, No. (%)**					0.13
No treatment	1 (50.0)	3 (30.0)	4 (66.7)	1 (5.6)	
EBRT	1 (50.0)	4 (40.0)	0 (0.0)	8 (44.4)	
VBT	0 (0.0)	1 (10.0)	0 (0.0)	5 (27.8)	
CTRT	0 (0.0)	2 (20.0)	2 (33.3)	4 (22.2)	

CTRT = combined adjuvant chemotherapy and radiotherapy; EBRT = external beam radiotherapy; EC = endometrial cancer; EDM = exonuclease domain mutations; EEC = endometrioid endometrial cancer; FIGO = International Federation of Gynecology and Obstetrics; LVSI = lymphovascular space invasion; MMRd = mismatch repair; POLEmut = POLE-ultramutated; PORTEC = Post Operative Radiation Therapy in Endometrial Carcinoma; VBT = vaginal brachytherapy.

Supplementary Table 2. Distribution of the Lynch syndrome associated second primary cancer types

2nd Primary cancers, No. (%)<br>Type, No. (%)	Methylated<br>15 (5.5)<br>*N*	Other<br>2 (2.9)<br>*N*	Lynch<br>4 (11.1)<br>*N*
Colon	5 (33.3)	2 (100)	3 (75.0)
Gallbladder	1 (6.7)	0 (0)	0 (0)
Kidney	1 (6.7)	0 (0)	0 (0)
Pancreas	2 (13.3)	0 (0)	0 (0)
Rectosigmoid	1 (6.7)	0 (0)	0 (0)
Rectum	2 (13.3)	0 (0)	0 (0)
Stomach, excl. cardia	2 (13.3)	0 (0)	0 (0)
Urinary bladder	1 (6.7)	0 (0)	0 (0)
Ureter	0 (0)	0 (0)	1 (25.0)

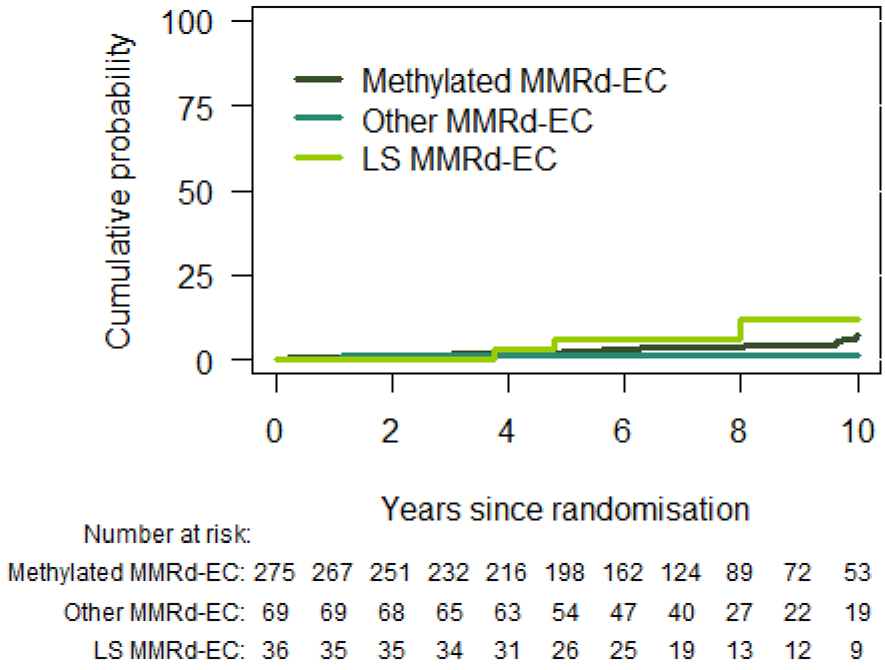

Supplementary Figure 2. Cumulative incidence of developing a subsequent Lynch syndrome associated primary cancer after a primary endometrial cancer.
